# Prevalence of Gastrointestinal Parasitic Infections and Associated Risk Factors Among Secondary School Students in Wonji Shoa, Adama District, East Shoa Zone, Oromia Region, Ethiopia

**DOI:** 10.1155/2024/5520924

**Published:** 2024-09-17

**Authors:** Abera Adugna, Tilahun Yohannes, Solomon Tesfaye

**Affiliations:** Department of Biology College of Natural and Computational Sciences University of Gondar, Gondar, Ethiopia

**Keywords:** gastrointestinal, prevalence, risk factors, school children, Wonji Shoa

## Abstract

Intestinal parasitic infections (IPIs) remain a significant contributor to morbidity and mortality globally, particularly in developing countries such as Ethiopia. Periodic assessments of IPI prevalence are essential prerequisite for effective control measures. Therefore, this cross-sectional study is aimed at determining the prevalence of gastrointestinal parasitic infections and associated risk factors among schoolchildren at Wonji Shoa Secondary School, East Shoa Zone, Adama district, Oromia region, Ethiopia, between January and June 2022. A simple random stratified sampling technique was employed to select participants. Sociodemographic and risk factor data were collected using a structured questionnaire. Stool samples were examined to identify parasites. Data were analyzed using SPSS version 20. Descriptive statistics, chi-square tests, and logistic regression were conducted to assess associations between variables and then the strength of the association. A *p* value < 0.05 was considered statistically significant. Of the 403 selected students, 330 completed the study that makes 81.89% response success. The overall IPI prevalence was 16.66% (55/330), with a higher prevalence among males (60%, 33/55) than females (40%, 22/55). Five parasite species were identified: two protozoa (*Entamoeba histolytica* and *Giardia lamblia*) with a combined prevalence of 9.70% (32/330) and three helminths (*Ascaris lumbricoides*, *Hymenolepis nana*, and *Taenia* spp.) with a combined prevalence of 6.97% (23/330). Cysts were detected in 62.5% of *E. histolytica* cases (15/24), and eggs were detected in 76.92% of *A. lumbricoides* cases (10/13). The study revealed a substantial IPI prevalence (16.66%) among the students. This finding underscores the need for effective prevention and control strategies. The predominance of parasitic infections among males is notable requiring further investigation of the factors. The identification of multiple parasite species indicates a complex epidemiological scenario. The presence of protozoan cysts and helminthic eggs highlights the potential for fecal-oral transmission and the importance of improved sanitation and hygiene practices.

## 1. Background of the Study

Human intestinal parasites remain a significant global health burden, particularly in developing countries such as Ethiopia, contributing substantially to morbidity and mortality [1]. Gastrointestinal parasitic infections (GIPIs) are prevalent worldwide, with a particularly high endemic rate in regions including Africa, Southeast Asia, South America, and Eastern Europe [2]. Intestinal parasites are categorized as either helminths (worms) or protozoa, both of which inhabit the human intestine [3]. Protozoa exist in two forms: trophozoites and cysts. Trophozoites are the active, reproductive stage capable of causing infection, while cysts are the dormant, resistant form transmitted through fecal-oral contamination [1]. Helminths are another primary causative agent of GIPIs and are classified as nematodes (roundworms), cestodes (tapeworms), and trematodes (flukes) [4]. Adult worms reside in the intestine and shed eggs or larvae through feces. Transmission primarily occurs via the fecal-oral route or through skin penetration [5].

The most prevalent protozoan intestinal parasites include *Entamoeba histolytica*, *Giardia lamblia*, *Balantidium coli*, and *Cryptosporidium parvum*, while common helminths comprise *Ascaris lumbricoides*, hookworm, *Hymenolepis nana*, *Taenia* spp., and *Schistosoma mansoni* [5]. A strong correlation exists between high GIPI prevalence and factors such as poverty, low income, inadequate sanitation, overcrowding, improper waste management, limited access to clean water, tropical climate, and low latitude [3, 6]. Globally, approximately 3.5 billion individuals are affected by GIPIs, resulting in over 200,000 annual deaths. Ethiopia alone accounts for 50,000 of these fatalities [7]. Rural populations and school-aged children are disproportionately impacted by high infection rates and intensities [8]. The latter group is particularly vulnerable due to behaviors such as playing in contaminated soil, poor hygiene practices, and consuming contaminated food and water [9].

Intestinal parasitic infections (IPIs) constitute a significant burden of disease, particularly among children in developing nations [10]. More than 267 million preschool-aged and over 568 million school-aged children reside in regions with intensive IPI transmission, necessitating effective treatment and prevention strategies [11]. Sub-Saharan Africa, including Ethiopia, exhibits a high prevalence of IPIs among schoolchildren [12], with substantial negative impacts on nutritional and cognitive development, school attendance, and dropout rates [8]. The consequences of IPIs extend beyond these immediate effects, encompassing cognitive impairment, malnutrition, anemia, increased susceptibility to other infections [13], malabsorption, vitamin and mineral deficiencies, chronic blood loss, diarrhea, organ damage, impaired mental development, and stunted growth, with children experiencing more severe effects than adults [14, 15].

Effective prevention and control of IPIs necessitate a comprehensive assessment of prevalence and the identification of associated risk factors, particularly within vulnerable populations [16]. The endemicity of IPIs in many regions is closely linked to behavioral, geographical, economic, and social conditions [17]. The study area exhibited several factors conducive to IPI transmission, including prevalent open defecation, low educational attainment, large family size, inadequate living conditions, scarcity of potable water, and a warm, humid climate. Notably, a high proportion of male students engaged in irrigation-related agricultural activities, often without proper footwear. This study is aimed at determining the prevalence of IPIs and identify associated risk factors within this context.

## 2. Materials and Methods

### 2.1. Description of the Study Area

**Figure 1:**
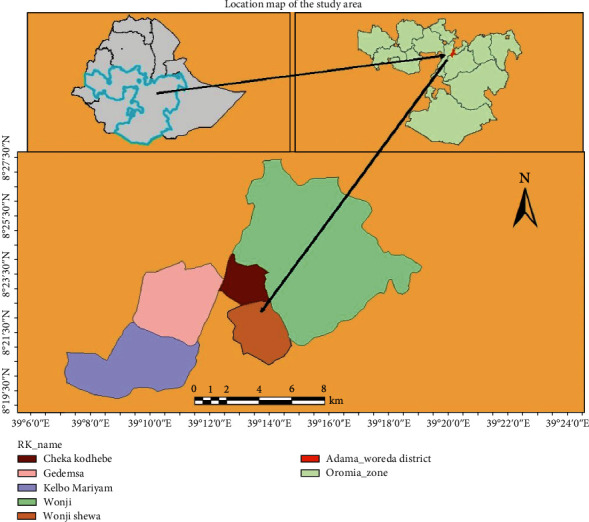
Map of the study area.

The study was conducted at Wonji Shoa Senior secondary school, located in East Shoa Zone, Oromia Region, Ethiopia. Its geographic coordinates are 8°30′ N latitude and 39°20′ E longitude, with an elevation of 1540 masl (Figure 1). The area experiences an average annual rainfall of 831 mm and mean maximum and minimum temperatures of 27°C and 15°C, respectively. Notably, the region's historical significance includes the establishment of Ethiopia's first modern sugar industry in 1954.

### 2.2. Research Design

A school-based cross-sectional study was conducted among secondary school students at Wonji Shoa School from January to June 2022 to determine the prevalence of and associated risk factors for IPIs.

### 2.3. Inclusion and Exclusion Criteria

Study participants consisted of students whose parents/guardians provided voluntary written consent and who had not taken any antiparasitic medication within the preceding 2 months. Students who had received antiparasitic treatment within the past 2 months or whose parents/guardians declined to provide written consent were excluded from the study.

### 2.4. Data Collection Methods

#### 2.4.1. Structured Questionnaire

Structured, closed-ended questionnaires were employed to gather sociodemographic and risk factor data. The research instrument was initially developed in English and subsequently translated into local languages, Afaan Oromo and Amharic. A pretest was conducted to ensure questionnaire quality prior to implementation in the study. A pretest was administered to 10% of participants at a separate school; however, these data were not incorporated into the study results.

#### 2.4.2. Parasitological Examination

Approximately 5 g of fresh stool specimens was collected from each school-aged child. Prior to collection, participants received detailed instructions on proper sample handling and were provided with plastic containers, waterproof paper, and applicator sticks for specimen transport. Stool examinations were conducted by laboratory technicians at Wonji Shoa Alemtena Health Center.

##### 2.4.2.1. Direct Wet Mount Technique

Direct wet mount parasitological examination was conducted at Wonji Shoa Alemtena Health Center. Approximately 1 g of stool sample was emulsified in separate drops of saline and iodine on clean glass slides. Coverslips were applied, and the prepared slides were examined under a light microscope at 10× and 40× magnifications to detect the ova, larvae, cysts, and trophozoites of intestinal parasites.

##### 2.4.2.2. Formal-Ether Concentration Method

Approximately 3 g of each stool sample was homogenized in 7 mL of 10% formalin and filtered through gauze. Subsequently, 3 mL of diethyl ether was added to the formalin solution, and the mixture was vigorously agitated for 30 sec by inverting the stoppered tube. The sample was then centrifuged at 2000 rpm for 3 min, resulting in four distinct layers. The upper three layers were discarded. The remaining sediment, containing the parasitic elements, was resuspended in normal saline and transferred to a microscope slide. The slide was examined under a microscope using a 10× objective lens followed by a 40× objective lens to identify cysts and ova.

### 2.5. Sample Size Determination Techniques

Sample size was calculated using the single population proportion formula as outlined by Charan and Biswas [18]. This method has been previously employed in similar studies conducted in Merawi elementary schools [17]. Given the absence of prior research on IPIs among Wonji Shoa secondary school children, a prevalence of 50% was assumed to maximize sample size. With a desired 95% confidence interval, a 5% margin of error, and an anticipated 5% nonresponse rate, the calculated minimum sample size was 403.

### 2.6. Sampling Technique

To select the study population, classes were initially chosen through a lottery method. A quota, proportional to the class size, was assigned to each selected class. Subsequently, participants were stratified based on sociodemographic characteristics, and then, participants were selected using simple random sampling.

### 2.7. Data Analysis

Data were analyzed using Statistical Package for Social Sciences (SPSS) version 20.0. Descriptive statistics, including frequencies and percentages, were computed for categorical variables. The association between the prevalence of IPIs and sociodemographic and associated risk factors was assessed using the chi-square test. Binary logistic regression was employed to determine the strength of association between variables, expressed as crude odds ratios. A *p* value less than 0.05 was considered statistically significant for all analyses.

### 2.8. Ethical Considerations

Prior to commencement, ethical approval for the study was secured from the Ethical Review Committee of the College of Natural and Computational Science, University of Gondar. Subsequent permissions were obtained from school principals and relevant authorities in the study area. Written informed consent was acquired from the students' parents/guardians prior to participation. Students were referred to a local health center for stool examination, and those diagnosed with parasitic infections received appropriate antiparasitic treatment as prescribed by physician. All costs associated with diagnosis and treatment were covered by the research team.

### 2.9. Quality Assurance

Research questionnaires were initially developed in English and subsequently translated into Afan Oromo and Amharic, the local languages. To ensure conceptual equivalence, back translation into English was performed. All diagnostic procedures and treatment were conducted by laboratory technicians and physicians at Wonji Shoa Clinic in accordance with standard operating procedures outlined by the World Health Organization. Data were meticulously cleaned, coded, and entered.

## 3. Results

### 3.1. Sociodemographic Characteristics of the Study Participants

A total of 403 participants were enrolled in the study, of whom 330 (81.89%) provided complete data and were included in the final analysis. The sample comprised 172 males (52.12%) and 158 females (47.87%). Regarding residence, 169 participants (51.21%) were from rural areas, while 161 (48.78%) were from urban areas. Based on grade level, the distribution was as follows: Grade 9 (12.72%), Grade 10 (21.81%), Grade 11 (40.90%), and Grade 12 (24.54%). The age of the participants ranged from 14 to 24 years, with the majority (244, 73.93%) falling within the 17–20 age group (Table 1).

### 3.2. Prevalence of GIPIs at Wonji Shoa Secondary School Children From January to June

**Figure 2:**
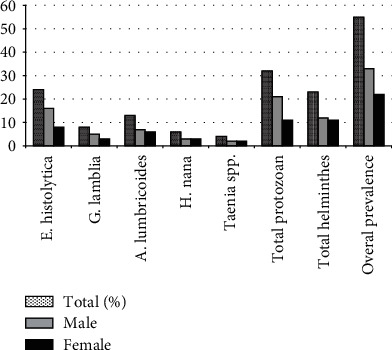
Prevalence of gastrointestinal parasitic infections among Wonji Shoa secondary school students (*n* = 330), January–June 2022.

Among the 330 school-aged children examined, 16.66% (55, *n* = 330) were diagnosed with at least one IPI. Protozoan parasites exhibited a higher prevalence (58.12%, *n* = 32) compared to helminths (41.82%, *n* = 23). A total of five IPI species were identified, including two protozoa and three helminths. *E. histolytica* was the most prevalent protozoan (7.27%, *n* = 24), while *A. lumbricoides* was the most common helminth (3.94%, *n* = 13) (Figure 2).

### 3.3. Different Developmental Stages of Parasitic Infections Identified Among School Children at Wonji Shoa Secondary School From January to June 2022

Of the 55 total infections, 32 (58.18%) were attributed to protozoan parasites. Among these, 20 (62.5%) were identified at the cyst stage, while 12 (37.5%) were identified at the trophozoite stage. Regarding helminth infections, which accounted for 23 (41.82%) of the total, 10 (76.92%) *A. lumbricoides*, 2 (33.33%) *H. nana*, and 3 (75.0%) *Taenia* spp. infections were detected by the identification of their respective eggs. Additionally, 4 (66.67%) *H. nana* and 1 (25.0%) *Taenia* spp. infections were diagnosed based on the detection of adult worms (Table 2).

### 3.4. Factors Associated With IPIs Among Wonji Shoa Secondary School Children

#### 3.4.1. Chi-Square Analysis of Sociodemographic Factors Associated With IPIs

Chi-square analysis revealed age group as the only statistically significant predictor of IPIs (*p* ≤ 0.00). Higher prevalence rates of IPIs were observed among males (60%), participants aged 14–16 years (56.36%), rural residents (63.64%), students in Grade 11 (52.73%), and those with a middle monthly income (60%) (Table 3).

### 3.5. Chi-Square Analysis of Risk Factors Associated With the IPIs

Chi-square analysis revealed no significant associations between the studied risk factors and the prevalence of IPIs. However, a notable trend emerged: individuals who regularly consumed boiled water exhibited a significantly lower prevalence of IPIs (9/55, 16.36%) compared to those who did not (46/55, 83.64%) (Table 4).

### 3.6. Logistic Regression Analysis (LRA) of the Risk Factors With IPIs Among School Children in Wonji Shoa Secondary School Children From January to June 2022

Univariate logistic regression analysis was conducted to assess the association between sociodemographic factors and other potential risk factors with IPIs (Table 5). Age was identified as a statistically significant predictor of IPIs (*p* < 0.25). Compared to other grade levels, students in Grade 12 were approximately two times more likely to be infected with IPIs (OR = 1.889–4.501, *p* ≤ 0.388). Additionally, individuals experiencing additional symptoms alongside diarrhea were found to be 2.9 times more likely to be infected compared to those without (*p* ≤ 0.392) (Table 5).

## 4. Discussion

Regular monitoring of GIPI trends within specific communities is essential for evaluating the effectiveness of existing prevention and control programs and informing the development of future therapeutic interventions. School-based deworming programs are a key component of community-wide GIPI control efforts and require ongoing assessment. A previous systematic review and meta-analysis by Legese et al. [9] reported a significant decline in GIPI prevalence among Ethiopian children over the past two decades, although the burden of infection remains substantial among school-aged populations. This study is aimed at assessing the prevalence and associated risk factors of GIPIs among students at Wonji Shoa secondary school, Ethiopia. The overall GIPI prevalence in this study was 16.66% (55/330), which is higher than the 12% reported among schoolchildren in Taif, Saudi Arabia [19].

The prevalence of GIPIs observed in this study is lower than that reported in several previous studies. For instance, Ayalew, Debebe, and Worku [20] found a prevalence of 79% among schoolchildren in Delgi, Ethiopia, while Alamir, Awoke, and Feleke [21] reported a rate of 77.9% in Dagi Primary School, Ethiopia. Other studies conducted in Ethiopia, Nepal, Iran, and Sudan have documented GIPI prevalence ranging from 19.9% to 79% [10, 17, 22–27]. These discrepancies in prevalence may be attributed to a multitude of factors, including variations in sanitation and hygiene practices, public health infrastructure, food and water safety, nutritional status, host immunity, access to safe water, socioeconomic conditions, and environmental determinants. Previous research has consistently emphasized the complex interplay of these elements in shaping GIPI prevalence [28].

The overall prevalence of protozoan parasitic infections in this study was 9.70%, which is comparable to the rate of 8.1% reported among primary school children in Sasiga district, Ethiopia [24]. However, this figure is lower than the prevalence observed in other studies, including 18.5% among school children in Itahari, Nepal [29]; 32.9% in Dakahlia governorate, Egypt [30]; and 29.53% in Merawi elementary schools [17]. These discrepancies may be attributed to differences in factors such as socioeconomic conditions, hygiene practices, and environmental factors influencing the transmission of protozoan parasites, primarily through the fecal-oral route, including contaminated hands, food, and water.

*E. histolytica* was the most prevalent intestinal parasite identified in this study, with a prevalence rate of 7.27%. This finding is lower than those reported in studies conducted in Delgi, Ethiopia (27%) [20]; Dakahlia governorate, Egypt (12.3%) [30]; Merawi elementary schools (19.10%) [17]; Aksum town, Northern Ethiopia (17.00%) [25]; Motta town, Ethiopia (17%) [5]; Bahir Dar Dona Berber primary school, Ethiopia (24.5%) [12]; and rural schools in Sohag Governorate, Egypt (20.4%) [31]. Conversely, the prevalence in the current study was higher than that observed in Debre Elias primary schools, Ethiopia (6.7%) [16], and Delo-Mena district, Ethiopia (5%) [32]. These variations in prevalence may be attributed to differences in handwashing practices, personal hygiene, and overall living conditions. Globally, the transmission of *E. histolytica* is commonly facilitated by contaminated hands, water, and food, as well as poor personal hygiene and environmental sanitation [33].

Helminth infections were detected in 6.96% of participants, a prevalence comparable to that reported among primary school children in Birbir town, Ethiopia (7.1%) [6]. *A. lumbricoides* was the most prevalent helminth, affecting 3.94% of participants, a rate higher than that observed among primary school children in Debre Elias, Ethiopia (0.67%) [16]. However, the current prevalence was lower than that reported in several other studies, including those conducted among Merawi elementary school students (6.4%) [17]; rural school children in Sohag Governorate, Egypt (6.5%) [31]; University of Gondar community school children (6.5%) [22]; primary school children in Birbir town (8.8%) [6]; Aksum town (9.0%) [25]; Bamo No. 2 primary school (12%) [34]; Sasiga district (22.7%) [24]; Shashamane town (24.1%) [35]; Lumame town (32.6%) [36]; Tilili town (39.7%) [37]; and Delgi (48%) [20]. Potential risk factors for *A. lumbricoides* infection include contact with contaminated soil, agricultural activities, and inadequate knowledge about transmission, poor personal and environmental hygiene, and insufficient hand washing practices.

The present study revealed a higher prevalence of IPIs among male participants compared to females. This finding aligns with the previous research conducted among schoolchildren in Chilga [38] and Delgi [20], both located in Ethiopia. Conversely, a study in Adigrat, Ethiopia, reported a higher prevalence among females [39]. Potential explanations for the observed gender disparity in IPI prevalence include differences in gender roles and behaviors. Male participants may be more exposed to contaminated soil and water through agricultural activities, a common source of IPI transmission. In contrast, females may exhibit greater attention to hygiene and environmental sanitation. Additionally, the higher physical activity levels and outdoor play among male students may increase their risk of exposure to infectious agents. These factors could contribute to the elevated IPI prevalence observed in the male population of the study area.

A statistically significant difference in overall IPI prevalence was observed across age groups. Higher infection rates were found in younger age brackets, specifically among students aged 1–16 years. This finding suggests that older children may have improved knowledge of hygiene practices, such as hand washing, compared to their younger peers. IPI prevalence was significantly higher among participants from rural compared to urban areas. This observation aligns with the previous research conducted in Merawi elementary schools [17], Motta town [5], Sasiga district [24], and Adigrat town [39]. Potential contributing factors to the elevated IPI prevalence in rural settings include suboptimal personal and environmental hygiene, inadequate hand washing practices, open defecation, and increased exposure to contaminated soil, water, and domestic animals.

## 5. Conclusion

The prevalence of IPIs among the study population was determined to be 16.66% (*n* = 55). Males exhibited a higher infection rate compared to females. Among the identified parasites, *E. histolytica* and *A. lumbricoides* were the most prevalent protozoan and helminth, respectively. Other parasites detected included *G. lamblia*, *H. nana*, and *Taenia* spp. Despite evaluation, no significant associations were found between IPI infection and the assessed risk factors. The majority of infections (36.36%) were diagnosed through cyst detection, while 23.64% were identified by egg examination. Notably, participants in the youngest age group, those from rural areas, and those with lower monthly family income demonstrated a higher prevalence of IPIs. It is recommended that community-based health education, mass drug administration, and school-based deworming programs be implemented consistently to address the burden of IPIs in the study area.

## Figures and Tables

**Table 1:** Sociodemographic characteristics of study participants at Wonji Shoa senior secondary school children from January to June 2022.

**Parameters**	**Category**	**F** **r** **e** **q** **u** **e** **n** **c** **y** = 330	**Percent (%)**
Sex	Male	172	52.12
	Female	158	47.87

Residence	Urban	161	48.78
	Rural	169	51.21

Age groups	14–16 years	64	19.39
	17–20 years	244	73.93
	21–24 years	22	6.66

Grade levels	Grade 9	42	12.72
	Grade 10	72	21.81
	Grade 11	135	40.90
	Grade 12	81	24.54

Family monthly income	< 1000 birr	67	20.30
	3000–5000 birr	199	60.30
	> 5000 birr	64	19.39

**Table 2:** Different developmental stages of IPIs identified during the diagnosis of school children at Wonji Shoa secondary school from January to June 2022.

**The life stages of IPIs detected during the diagnosis**			
**Protozoan**	**Total identified parasite, ** **n** = 55** (%)**	**Developmental stages of the parasite**	
		**Trophozoites**	**Cyst stage**
*E*. *histolytica*	24 (43.64)	9 (37.5)	15 (62.5)
*G*. *lamblia*	8 (14.55)	3 (37.5)	5 (62.5)
Total protozoan (%)	32 (58.18)	12 (37.5)	20 (62.5)
Helminths	Total (%)	Eggs	Larval/adult
*A. lumbricoides*	13 (23.64)	10 (76.92)	3 (23.08) adults
*H*. *nana*	6 (10.91)	2 (33.33)	4 (66.67) adults
*Taenia* spp	4 (7.27)	3 (75.0)	1 (25.0) adult
Total helminths (%)	23 (41.82)	15 (65.22)	8 (34.78)

**Table 3:** Sociodemographic characteristics of the study subjects in Wonji Shoa secondary school children from January to June 2022.

**Risk factors**	**Category**	**Frequency (%)**	**Positive (%) (** **n** = 55**)**	**Negative (%) (** **n** = 275**)**	**χ** ^**2**^	**p** ** value**
Sex	Male	172 (52.12)	33 (60.00)	139 (50.55)	1.642	0.2000
	Female	158 (47.87)	22 (40.00)	136 (49.45)		

Age	14–16	64 (19.39)	31 (56.36)	33 (12.00)	80.6	0.000∗
	17–20	244 (73.93)	14 (25.45)	230 (83.64)		
	21–24	22 (6.66)	10 (18.18)	12 (4.36)		

Residence	Urban	161 (48.78)	20 (36.36)	141 (51.27)	2.880	0.410
	Rural	169 (51.21)	35 (63.64)	134 (48.73)		

Grade level	9	42 (12.72)	7 (12.73)	35 (12.73)	0.410	0.522
	10	72 (21.81)	9 (16.36)	63 (22.91)		
	11	135 (40.90)	29 (52.73)	106 (38.55)		
	12	81 (24.54)	10 (18.18)	71 (25.82)		

Family monthly income	< 1000	67 (20.30)	14 (25.45)	53 (19.27)	1.664	0.435
	3000–5000	199 (60.30)	33 (60.00)	166 (60.36)		
	> 5000	64 (19.39)	8 (14.55)	56 (20.36)		

^∗^Statistically significant association with age.

**Table 4:** Associated risk factors with IPIs among school children at Wonji Shoa secondary school from January to June 2022.

**Risk factors**	**Category**	**Frequency (%)**	**Positive (%) (55)**	**Negative (%) (275)**	**χ** ^**2**^	**p** ** value**
The source of drinking water?	River	83 (25.15)	9 (16.36)	74 (26.91)	3.377	0.337
	Ground	98 (29.69)	17 (30.91)	81 (29.45)		
	Tap	80 (24.24)	14 (25.45)	66 (24.0)		
	Spring	69 (20.90)	15 (27.27)	54 (19.64)		

The way of treating drinking water?	Boiling	57 (17.27)	9 (16.36)	48 (17.45)	0.103	0.748
	Not boiling	273 (82.72)	46 (83.64)	227 (82.55)		

Had washing habits before a meal?	Yes	280 (84.84)	47 (85.45)	233 (84.73)	0.810	0.368
	No	50 (15.15)	8 (14.55)	42 (15.27)		

Is there any toilet in your garden?	Yes	246 (74.54)	45 (81.82)	201 (73.09)	1.840	0.175
	No	84 (25.45)	10 (18.18)	74 (26.91)		

Is there any toile in your School?	Yes	273 (82.72)	48 (87.27)	225 (81.82)	0.954	0.329
	No	57 (17.27)	7 (12.73)	50 (18.18)		

Type of latrine in the garden	Open field	124 (37.57)	20 (36.36)	104 (37.82)	0.041	0.839
	Latrine	206 (62.42)	35 (63.64)	171 (62.18)		

Habits of using raw meat	Yes	191 (57.87)	32 (58.18)	159 (57.82)	0.002	0.960
	No	139 (42.12)	23 (41.82)	116 (42.18)		

Have you ever used raw vegetable	Yes	104 (31.51)	19 (34.55)	85 (30.91)	0.062	0.803
	No	226 (68.48)	36 (65.45)	190 (69.09)		

Is there any symptom of diarrhea?	Yes	46 (13.93)	4 (7.27)	42 (15.27)	2.445	0.118
	No	284 (86.06)	51 (92.73)	233 (84.73)		

If any, what type of diarrhea is it?	Normal	218 (66.80)	36 (65.45)	182 (66.18)	1.795	0.408
	Mucoid	40 (12.12)	4 (7.27)	36 (13.09)		
	Watery	47 (14.24)	11 (20.0)	36 (13.09)		
	Bloody	25 (7.57)	4 (7.27)	21 (7.64)		

Additional symptoms	Abdominal pain	127 (38.48)	17 (30.91)	110 (40.0)	4.919	0.178
	Vomiting	30 (9.09)	9 (16.36)	21 (7.64)		
	Nausea	70 (21.21)	11 (20.0)	59 (21.45)		
	Appetite	103 (31.21)	18 (32.73)	85 (30.91)		

Habits of cutting finger nails	Yes	281 (85.15)	48 (87.27)	233 (84.73)	0.702	0.402
	No	49 (14.84)	7 (12.73)	42 (15.27)		

Family monthly salary	< 1000	67 (20.30)	14 (25.45)	53 (19.27)	1.664	0.435
	3000–5000	199 (60.30)	33 (60.0)	166 (60.36)		
	> 5000	64 (19.39)	8 (14.55)	56 (20.36)		

Ways of household waste disposal	Burning	88 (26.66)	14 (25.45)	74 (26.91)	1.664	0.435
	Buried	99 (30.00)	18 (32.73)	81 (29.45)		
	Randomly	143 (43.33)	23 (41.82)	120 (43.64)	0.235	0.889

**Table 5:** Associated risk factors with the prevalence of IPIs among Wonji Shoa secondary school, East Shoa, Ethiopia, from January to June 2022.

**Associated risk factors**	**Categories**	**Frequency (%)**	**Positive (%) (55)**	**Negative (%) (275)**	**COR CI 95%**	**p** ** value**
Sex	Male	172 (52.12)	33 (60.00)	139 (50.55)	0.202 [0.814–2.645]	1.468
	Female	158 (47.87)	22 (40.00)	136 (49.45)	1	

Age	14–16	64 (19.39)	31 (56.36)	33 (12.00)	0.073 [0.027–0.198]	0.000^∗^
	17–20	244 (73.93)	14 (25.45)	230 (83.64)	1.127 [0.427–2.979]	
	21–24	22 (6.66)	10 (18.18)	12 (4.36)	1	

Grade level	9	42 (12.72)	7 (12.73)	35 (12.73)	1.600 [0.550–4.561]	0.388
	10	72 (21.81)	9 (16.36)	63 (22.91)	1.600 [0.631–4.651]	
	11	135 (40.90)	29 (52.73)	106 (38.55)	2.000 [0.889–4.501]	
	12	81 (24.54)	10 (18.18)	71 (25.82)	1	

Residence	Urban	161 (48.78)	20 (36.36)	141 (51.27)	1.208 [0.677–2.158]	0.522
	Rural	169 (51.21)	35 (63.64)	134 (48.73)	1	

Family monthly salary	< 1000	67 (20.30)	14 (25.45)	53 (19.27)	1.848 [0.718–4.764]	0.440
	3000–5000	199 (60.30)	33 (60.00)	166 (60.36)	1.392 [0.6–3.190]	
	> 5000	64 (19.39)	8 (14.55)	56 (20.36)	1	

Source of drinking water	River	83 (25.15)	9 (16.36)	74 (26.91)	0.438 [0.178–1.074]	0.478
	Ground	98 (29.69)	17 (30.91)	81 (29.45)	0.756 [0.348–1.640]	
	Tap	80 (24.24)	14 (25.45)	66 (24.0)	0.764 [0.339–1.721]	
	Spring	69 (20.90)	15 (27.27)	54 (19.64)	1	

How to use drinking water?	By boiling	57 (17.27)	9 (16.36)	48 (17.45)	0.880 [0.405–1.916]	0.810
	Not boiling	273 (82.72)	46 (83.64)	227 (82.55)	1	

Hand washing habit before a meal?	Yes	280 (84.84)	47 (85.45)	233 (84.73)	1.514 [0.610–3.753]	0.371
	No	50 (15.15)	8 (14.55)	42 (15.27)	1	

Toilet in the grade	Yes	246 (74.54)	45 (81.82)	201 (73.09)	1.657 [0.794–3.456]	0.178
	No	84 (25.45)	10 (18.18)	74 (26.91)	1	

Is there any toilet in the school?	Yes	273 (82.72)	48 (87.27)	225 (81.82)	1.524 [0.651–3.566]	0.331
	No	57 (17.27)	7 (12.73)	50 (18.18)	1	

Type of latrine in the garden	Open field	124 (37.57)	20 (36.36)	104 (37.82)	0.0940 [0.515–1.714]	0.839
	Latrine	206 (62.42)	35 (63.64)	171 (62.18)	1	

Raw meat	Yes	191 (57.87)	32 (58.18)	159 (57.82)	1.051 [0.564–1.825]	0.960
	No	139 (42.12)	23 (41.82)	116 (42.18)	1	

Habits of consuming raw vegetable	Yes	104 (31.51)	19 (34.55)	85 (30.91)	0.803 [0.514–1.674]	0.803
	No	226 (68.48)	36 (65.45)	190 (69.09)	1	

Symptoms of diarrhea	Yes	46 (13.93)	4 (7.27)	42 (15.27)	0.435 [0.149–1.268]	0.127
	No	284 (86.06)	51 (92.73)	233 (84.73)	1	

Type of diarrhea	Normal	218 (66.80)	36 (65.45)	182 (66.18)		0.413
	Mucoid	40 (12.12)	4 (7.27)	36 (13.09)	1.963 [0.314–2.956]	
	Watery	47 (14.24)	11 (20.0)	36 (13.09)	1.604 [0.453–5.681]	
	Bloody	25 (7.57)	4 (7.27)	21 (7.64)		

Additional symptoms	Abdominal pain	127 (38.48)	17 (30.91)	110 (40.0)	0.730 [0.355–1.500]	0.392
	Vomiting	30 (9.09)	9 (16.36)	21 (7.64)	2.924 [0.797–5.139]	
	Nausea	70 (21.21)	11 (20.0)	59 (21.45)	0.880 [0.388–2.000]	
	Appetite lose	103 (31.21)	18 (32.73)	85 (30.91)	1	

Parental salary	< 1000	67 (20.30)	14 (25.45)	53 (19.27)	1.849 [0.718–4.764]	0.440
	3000–5000	199 (60.30)	33 (60.0)	166 (60.36)	1.392 [0.607–3.190]	
	> 5000	64 (19.39)	8 (14.55)	56 (20.36)	1	

Ways of waste disposal	Burning	88 (26.66)	14 (25.45)	74 (26.91)	0.987 [0.478–2.038]	0.972
	Buried	99 (30.00)	18 (32.73)	81 (29.45)	1.159 [0.588–2.284]	
	Randomly	143 (43.33)	23 (41.82)	120 (43.64)	1	

Abbreviations: 1 = reference point, COR = crude odd ratio, CI = confidence interval.

^∗^Statistically significant at *p* value < 0.05.

## Data Availability

The complete raw data for this study are in the possession of the authors and are available upon a reasonable request.
